# Is the binge-eating disorder a circadian disorder?

**DOI:** 10.3389/fnut.2022.964491

**Published:** 2022-07-22

**Authors:** Santiago A. Plano, Sebastián Soneira, Camila Tortello, Diego A. Golombek

**Affiliations:** ^1^Institute for Biomedical Research (BIOMED), National Scientific and Technical Research Council (CONICET), Catholic University of Argentina (UCA), Buenos Aires, Argentina; ^2^Laboratorio de Cronobiología, Universidad Nacional de Quilmes/CONICET, Buenos Aires, Argentina; ^3^Sección de Trastornos de la Conducta Alimentaria y Psiquiatría Nutricional, Servicio de Psiquiatría, FLENI, Buenos Aires, Argentina; ^4^Escuela de Educación, Universidad de San Andrés, Buenos Aires, Argentina

**Keywords:** binge-eating, circadian, sleep, chronotype, BED, NES

## Introduction

There is a wide range of eating disorders (EDs) which span from restrictive eating patterns such as anorexia nervosa, to the compulsive eating spectrum such as bulimia nervosa (BN), night-eating syndrome (NES), and binge-eating disorder (BED), all with complex multifactorial pathogenesis including biological, environmental, and psychological factors. In general, EDs enclosed within the compulsive spectrum include disordered bouts of feeding at different times of the day. Many aspects of these disorders are modulated by the circadian system, such as meal timing, mood, compulsive behavior, and sleep quality.

We conducted a systematic search combining binge eating studies with circadian/sleep analyses to assess the main aspects of the BED and its relationship with the circadian system. This was conducted on the electronic database MEDLINE/PubMed using the search string (binge eating) AND [(circadian) or (rhythm)]. We searched the literature references to find additional articles. We kept only the papers that treat binge eating as a disorder and not as a component of other disorders, such as the binge eating episodes in bulimia nervosa. In view of the scarce literature on the subject, here we propose a call to action for examining circadian patterns in more detail within BED.

## Binge eating disorder (BED)

Binge eating disorder (BED) is the most frequent eating disorder, affecting 1–3% of the population, with onset between 15.5–27.2 years of age and an average duration of 4–8 years (1, 2). Like most eating disorders, BED is more common in women (3.5%) vs. men (2.0%) (2). Despite being the most common eating disorder, many patients fail to receive a proper diagnosis or do. Indeed, in a representative sample of American adults, only 3.2% of respondents who met DSM-5 criteria for BED had received an appropriate formal diagnosis in the previous 12 months (3). Originally described by Spitzer (4), BED is characterized by the presence of recurrent binge-eating episodes with a minimum duration of 3 months and a frequency of > once a week. Another important fact to consider is the unpleasant nature of binge eating and the absence of purging behaviors or subsequent compensation.

An episode of binge eating is characterized by the ingestion of more food than what most people would eat in a similar period and circumstances, as well as a feeling of lack of control over what is ingested. Its clinical description represents a specific eating disorder, different from other mental disorders after its inclusion in 2013 in DSM-5 (5). The differential diagnoses of this disorder include Bulimia Nervosa (BN), the binge-eating and purging subtype of Anorexia Nervosa (BP-AN), and night eating syndrome (NES).

In BN, episodes of binge eating are usually followed by self-induced purging behaviors (generally vomiting). In BP-AN, the same thing happens, but body weight is noticeably decreased due to the intense restriction of meals. In both disorders, an intense fear of gaining weight is clearly present. Finally, the NES is characterized by recurring episodes of food intake at night, manifested by eating when waking up during the night or by excessive consumption of food after dinner (6). Clearly, in the NES the most important factor is the time of day in which the ingestion occurs. Described in the 1950s, the chronobiological aspects of NES have received more consideration: NES patients tend to express evening chronotype with a higher frequency of insomnia and overweight conditions (41, 42).

Patients with BED present a high prevalence of psychiatric and physical comorbidities. Between 30.0 and 80.0% of individuals with BED present lifetime comorbid mood and anxiety disorders (7, 8). Other common comorbidities reported in individuals with BED comprise numerous addiction disorders such as substance use/abuse (22.0%) (9), gambling problems (5.7–18.7%) (10) as well as compulsive buying (7.4–18.5%) (11).

The prevalence of binge eating disorder in individuals with obesity attending weight loss programs was found to be between 16–52% (12).

Individuals with obesity and comorbid eating disorders are at high risk of several medical and psychosocial complications such as diabetes, hypertension, and chronic pain (1, 13). A study with 152 treatment-seeking individuals with obesity found that those with binge eating disorder had higher BMIs, more severe levels of depression and obsessive-compulsive symptoms, and stronger feelings of inadequacy and inferiority than those without binge eating disorder (14).

## Chronotype and chrono-nutrition

Humans differ in their preferences for activity and sleep patterns during the day, reflecting interindividual differences in their daily physiological organization, defining morning and evening chronotypes (15, 16).

There is a clear association between mood and chronotypes (17, 18). In particular, the eveningness dimension could be considered a vulnerability factor to depressive symptoms and Major Depressive Disorder (19).

Different studies investigated the relationship between personality traits and chronotypes (20); evening types were associated with anxious, hostile, impulsive and depressive personality traits, while a morning chronotype was associated with traits such as conscientiousness and with the tendency to be compliant (21, 22).

The term chrono-nutrition refers to energy distribution processes including feeding-fasting rhythm, meal frequency, the duration of the eating period, and the relationship with metabolic health (23, 24). Meal regularity establishes the pattern of energy intake and distribution, which is crucial for health outcomes. On the other hand, meal irregularity, defined as food consumption at different times and in varying amounts throughout the day (25), is associated with obesity and metabolic-related disease (26). Several recent studies indicate that food intake at later times is highly associated with increased adiposity, obesity, and metabolic risk (26, 27). Moreover, eating a large amount of food during the evening increased the odds of obesity and metabolic syndrome (28). An increased ratio of evening-to-morning meals has been associated with an increase in the body mass index (BMI), while a higher morning-to-evening ratio has the opposite effect (29–31). The conclusion is that eating when the body is not ready to manage a large amount of energy (i.e., when we are preparing to sleep), has a great impact on our health (28). The proximity of food consumption to the nocturnal rise of melatonin was associated with impaired glucose homeostasis and increased adiposity (32, 33). Eating behavior has been linked to circadian rhythms (34), since clock genes may synchronize not only the feeding-fasting rhythm but also metabolism itself (35, 36). In this sense, the individual differences in chronotype could influence eating behavior as a zeitgeber (37). For instance, late chronotype or late mealtime were associated with an increased desire for high-fat foods and more appetite (38).

The relationship between the circadian system and specific eating disorders remains unclear in part, this may be due to nosographic aspects related to the diagnostic systems of EDs, particularly regarding the differential diagnoses between BED and NES.

Although NES is described as an eating disorder in the DSM, many authors have questioned its validity as a nosographic construct (39, 40), even when it represents the most relevant results associated with eating at night and its consequences. Individuals with BED or NES share the feeling of a loss of control over food consumption, but both syndromes differ in their timing. Several studies demonstrate that energy intake in NES patients tended to occur during the night, affecting the sleep-wake cycle (41, 42). In contrast, in BED patients, the available literature fails to systematically collect and inform the time of the binge behavior that may occur at any time of the day (43, 44). Only a few studies report circadian preferences in patients with eating disorders (37, 40, 45). A circadian approach to the BED indicated that subjects with either BED or NES were more likely to have an evening chronotype (40), as well as higher rates of anxious and depressive symptoms. Subjects with NES have similar sleep onset and offset times to those of controls (46, 47), suggesting that their delay in chronotype may be due to their eating behavior and not to the circadian system. Indeed, more work is needed to examine when binge episodes occur and what is their relationship with the feeding-fasting rhythm, the subject's chronotype. Social aspects should also be considered, e.g., if the binge occurs when people are alone, or if there is some preference for binge during week-days or during weekends which can contribute to social jetlag, another circadian disruption with metabolic consequences. In this sense, both NES and BED can act as a promotor of- as well as a consequence of- circadian disruption, inducing a vicious cycle between both alterations.

## Binge behavior and sleep

Sleep disturbances has been studied in patients with NES, which tend to present insomnia and periodic limb movements. Moreover, night eaters reported higher ratings of sleep perturbations and use of sleep medications (41). However, NES was not associated with day-time sleepiness. A rest-activity pattern study found no differences on sleep-onset time or total sleep duration in NES, although more awakenings and later sleep offset time have been reported (41).

A study of the rest-activity pattern in patients with BED demonstrated some alterations in their circadian behavior. BED patients exhibited a lower MESOR (mean estimate of circadian rhythm, derived from fitting the data to a cosine wave) and amplitude than control subjects (48). Patients also presented low sleep-efficiency values, but this was also present in an obese control group, indicating that it could be ascribed to overweight conditions (45). Different studies analyzing obese patients with and without BED confirmed this finding: sleep architecture abnormalities in patients with BED were also found in the obese control group (45). Despite this, another study demonstrated that BED patients show more minutes of wakefulness during the sleep period than normal-weight controls (49), presumably due to their binge episodes. In addition, significantly higher sleep-disruption parameters were found in children with BED than in obese non-BED and normal-weight controls (49). More work needs to be done to clarify the relationship between BED and the circadian pattern of sleep, sleep quality, and its dependence on obesity.

## Addictive-like eating behaviors

Binge eating disorder is recognized as an eating disorder involving compulsive food intake. Some authors point out that this type of behavior could be included within a theoretical construct called Food Addiction (FA) (50, 51), employed to describe addictive-like compulsive overeating which involves cravings and difficulties in abstaining from high-calorie foods (52). Even though FA could be present in patients with BED, it is important to note that while BED is a classified and diagnosable mental disorder, FA remains a controversial concept that has not yet been recognized as a diagnostic entity. FA shows some similarities with substance abuse disorders; indeed, both drug consumption and excessive food intake share neural changes in the brain reward system (53–56). Dopaminergic activity disruption appears to be a common root for drug intake and compulsive eating, supporting the food addiction concept (57).

As we mentioned, feeding is rhythmically distributed over a 24h period (58). Interestingly, a circadian rhythm for caloric intake has also been described (59–61); and, in accordance, a binge eating behavior rhythm was found in rats with an increase in dopamine receptor density in the nucleus accumbens (62). This “caloric preference” rhythm is regulated by the circadian system (59, 63–65), while its hedonic component depends on the rhythmic activity of DA, which in rodents peaks at night, corresponding with their nocturnal phase of activity and food consumption (66, 67). This DA release in the striatum depends on the circadian-controlled VTA rhythmic activity (68, 69). Indeed, more information is needed on the relationship between feeding and reward rhythms in humans.

There is also a correlation between chronotype and food preference: an evening chronotype is correlated with greater consumption of high calorie-beverages, caffeine, alcohol, nicotine, and fast food (70–72), and lower consumption of fruit, vegetables, and fish (73). Moreover, subjects with an evening type show a higher energy intake at night, and this effect was even larger during weekends (71, 74). The metabolic and hedonic regulation of feeding is rhythmic and depends on the circadian system; the timing of food consumption is crucial for the regulation of food intake, and the loss of this circadian pattern might lead to disorders that display compulsive eating, obesity, and metabolic syndrome as a part of its symptomatic complex (75).

## Conclusion

In this work, we focused on the literature exploring the main physiological aspects controlled by the circadian system that affect or are affected by the binge eating disorder, and how the circadian system may play a significant role in the development of more severe outcomes. We summarize the current state of the literature studying the circadian aspects of BED, to find out that is a topic yet to be explored and offer our suggestions for future research in BED to include designs capable of collecting sufficient and necessary information to study the role of the circadian system on the BED progression. The circadian system modulates different aspects of mood and mood-related behavior, including emotion, compulsive behavior, and regulatory control (76). All these features are in close relationship with BED and should be studied from a circadian perspective. Given the higher prevalence of BED in women, it would also be important to delve into the influence of neurohormonal systems in the genesis of the disorder and its relationship with biological rhythms.

One of the main difficulties to study circadian aspects of BED is the differential diagnostic overlapping between BED and NES. Food is a strong signal for the circadian system; both BED and NES alter the feeding-fasting rhythm causing a circadian disruption which, in turn, will affect feeding, creating a vicious cycle of important consequences for the patient's well-being. In this sense NES and BED can be consequence of- or promotor of- a circadian disruption and an evening chronotype. Future studies need to address the crosstalk between BED, NES and the circadian system, including some objective measures of sleep-wake cycle, circadian status and energy consumption, as well as some subjective measures to cover aspects related to sleep and chronotype. In this sense, the mechanisms behind binge timing, mood, compulsive behavior, and sleep alteration need to be acknowledged to create a more accurate BED vs. NES diagnosis.

## Author contributions

All authors listed have made a substantial, direct, and intellectual contribution to the work and approved it for publication.

## Funding

The studies in authors' laboratories were funded by the Universidad Nacional de Quilmes, CONICET and the National Science Agency (ANPCyT), Argentina.

## Conflict of interest

The authors declare that the research was conducted in the absence of any commercial or financial relationships that could be construed as a potential conflict of interest.

## Publisher's note

All claims expressed in this article are solely those of the authors and do not necessarily represent those of their affiliated organizations, or those of the publisher, the editors and the reviewers. Any product that may be evaluated in this article, or claim that may be made by its manufacturer, is not guaranteed or endorsed by the publisher.
